# The Effect of Flame-Retardant Additives DDM-DOPO and Graphene on Flame Propagation over Glass-Fiber-Reinforced Epoxy Resin under the Influence of External Thermal Radiation

**DOI:** 10.3390/molecules28135162

**Published:** 2023-07-01

**Authors:** Oleg P. Korobeinichev, Egor A. Sosnin, Artem A. Shaklein, Alexander I. Karpov, Albert R. Sagitov, Stanislav A. Trubachev, Andrey G. Shmakov, Alexander A. Paletsky, Ilya V. Kulikov

**Affiliations:** 1 Voevodsky Institute of Chemical Kinetics and Combustion SB RAS, 630090 Novosibirsk, Russia; e.sosnin@g.nsu.ru (E.A.S.); rasagitov@gmail.com (A.R.S.); satrubachev@gmail.com (S.A.T.); shmakov@kinetics.nsc.ru (A.G.S.); paletsky@kinetics.nsc.ru (A.A.P.); kulikovsas2001@gmail.com (I.V.K.); 2 Department of Physics, Novosibirsk State University, 630090 Novosibirsk, Russia; 3 Udmurt Federal Research Center, 426067 Izhevsk, Russia; shaklein@udman.ru (A.A.S.); karpov@udman.ru (A.I.K.)

**Keywords:** glass-fiber-reinforced epoxy resin, flammability, fire retardancy, phosphorus-containing flame retardant, graphene, limiting oxygen index, cone calorimetry, numerical simulation

## Abstract

The flammability of various materials used in industry is an important issue in the modern world. This work is devoted to the study of the effect of flame retardants, graphene and DDM-DOPO (9,10-dihydro-9-oxa-10-phosphaphenanthrene-10-oxide-4,4′-diamino-diphenyl methane), on the flammability of glass-fiber-reinforced epoxy resin (GFRER). Samples were made without additives and with additives of fire retardants: graphene and DDM-DOPO in various proportions. To study the flammability of the samples, standard flammability tests were carried out, such as thermogravimetric analysis, the limiting oxygen index (LOI) test, and cone calorimetry. In addition, in order to test the effectiveness of fire retardants under real fire conditions, for the first time, the thermal structure of downward flame propagation over GFRER composites was measured using thin thermocouples. For the first time, the measured thermal structure of the flame was compared with the results of numerical simulations of flame propagation over GFRER.

## 1. Introduction

Composite materials based on epoxy resin are promising materials for various industries, such as aviation, mechanical engineering, shipbuilding, etc. [1]. These materials have gained great popularity due to their high strength, flexibility, chemical resistance and thermal-insulation properties, together with their low specific weight. However, epoxy resin is a flammable material [2,3]; therefore, increased fire-resistance requirements are applied to composites based on it. When exposed to an external heat source, the combustibility of these materials can increase even more, which makes the issue especially important for products installed near a heat source (aircraft nacelles, car radiator mounts, etc.).

A prevalent way to improve the fire resistance of epoxy resin is to add fire retardants. Previously, halogen compounds have been used as epoxy-polymer flame-retardant additives for a long time. However, halogen-containing compounds, when burned, emit ac-rid and toxic smoke, which is harmful to humans and the environment [4,5]. Numerous studies have been carried out to find an alternative to halogen-containing flame retardants, and as a result, a switch to phosphorus-containing flame retardants has been proposed. Phosphorus-based additives such as 9,10-dihydro-9-oxa-10-phosphafenentren-10-oxide (DOPO) and its derivatives [6,7] can serve as an example. Moreover, some studies show that polymer composites containing both phosphorus and nitrogen compounds show a significant improvement in flame-retardant performance [8,9,10,11,12]. Therefore, due to their special structure, DOPO-based phosphonamidates can be effective flame retardants [13,14,15]. So, a fire retardant such as DDM-DOPO [16] can be used in a smaller amount as part of an epoxy binder, which favorably affects the physical and mechanical properties of fiberglass-reinforced plastics made from such materials.

Nano-material additive, such as graphene, has recently become another popular halogen-free flame retardant due to its ability to increase the formation of char on the surface of the polymer and to strengthen it due to graphitization [17,18]. Graphene nanoparticles can effectively reduce the melt flow and inhibit the flammable drips of epoxy resin during combustion, which generally prevents the spread of flames [19]. In [20] it was shown that graphene has adsorbing properties, so it can capture volatile combustible compounds on the sample surface, which also increases the fire resistance of the material.

To assess the combustibility of polymeric materials, usually thermogravimetric analysis (TGA), a UL-94 test, limiting oxygen index (LOI), and cone calorimetry are used. Often, the fire resistance of materials is evaluated only on the basis of these methods [7,21]. However, these tests do not always provide reliable information about the behavior of the material under fire conditions [22]. In this case, more accurate information can be obtained from experimental studies of flame propagation over the surface of the material. However, hardly flammable materials such as reinforced composites do not support combustion under standard atmospheric conditions [23,24]. Such composites can be studied under conditions of high oxygen concentration [25], in the presence of external thermal radiation [26] or in the presence of an external flame source [22].

In 1970–1980, Williams [27], Fernandez-Pello [28,29], and Hirano [30] et al. performed experimental studies of flame propagation over a solid surface under the action of an external heat flux. Work [31] studied the horizontal flame propagation over thermoplastic polymers: polymethyl methacrylate (PMMA), polypropylene (PP) and polystyrene (PS) plates, under the action of one-sided heating, and it was shown that the effect of sample thickness on the nature of flame propagation is less pronounced than the effect heat flux of external radiation. The same effect is also shown in [32]. Moreover, the application of additional heat flux caused the flame to propagate more quickly over PMMA slabs. The qualitative influence of the heat flow was observed during the combustion of wood-based materials, which do not allow the flame to spread to the upper part of the sample, but allow it to spread in the presence of an external heat flow. In addition, preheating the particle board samples (with a 2.2 kW/m^2^ radiant flux) increased the surface temperature by more than 100 °C, and therefore required less time and energy to reach the pyrolysis temperature. The preheated sample showed significantly faster flame propagation than the sample that was shielded prior to ignition. It was shown in [33] that under the influence of an increasing external heat flux, the rate of flame propagation of polyurethane foam and expanded polystyrene demonstrate different trends. The rate of flame propagation over polyurethane foam increases all the time, and fluctuates over expanded polystyrene. In [34], the thermomechanical analysis of PMMA slabs was carried out under the action of a powerful heater installed on one side of the sample. Most investigations on flame propagation under external thermal radiation source conditions are devoted to such polymers as PMMA, PP, etc. However, a relatively small number of works are devoted to flame propagation over the surface of composites based on epoxy resin (ER). For example, in work [25], the authors studied downward flame propagation over the surface of carbon-fiber-reinforced epoxy resin at various oxygen concentrations. In addition, there are no data on flame propagation over reinforced composites with the addition of fire retardants, with the exception of a small number of works [22]. To predict the behavior of polymeric materials in different fire scenarios, a number of numerical models were developed and tested for the purpose of comparison with the experimental data [35,36]. Meanwhile, little attention has been paid to development of models of flame propagation over reinforced polymers and polymers with the addition of flame retardants, and there are no widely accepted approaches to the theoretical description of the pyrolysis and combustion of inhibited polymers.

Thus, the purpose of this work was to study the effect of flame retardants DDM-DOPO and graphene on flammability and downward flame propagation over glass-fiber-reinforced epoxy resin (GFRER) under the action of external thermal radiation and to develop an effective model that would predict the flame propagation behavior for GFRER composites.

## 2. Results and Discussion

### 2.1. TGA, DTG, UL-94 HB, LOI Results

**Figure 1:**
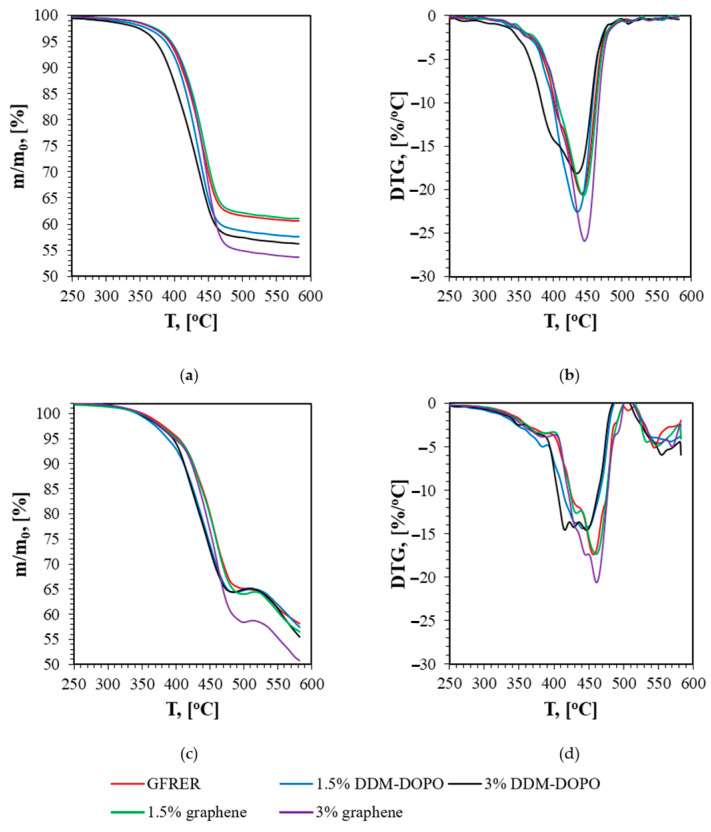
Thermogravimetric analysis data for GFRER with and without additives (**a**) TG data in Ar, (**b**) DTG data in Ar, (**c**) TG data in 79 v% He + 21 v% O_2_ and (**d**) DTG data in in 79 v% He + 21 v% O_2_.

Figure 1 shows the data of thermogravimetric analysis in inert atmosphere (Ar) for GFRER composites. It can be seen in Figure 1a that the addition of flame retardants reduces the amount of residue at the temperature of 580 °C, indicating an increase in the yield of pyrolysis products into the gas phase in the case of samples inhibited by flame retardants. Addition of 1.5% DDM-DOPO leads to a decrease in the residue mass by ~3% relative to the sample without the additives, while the addition of 3% DDM-DOPO leads to a decrease in the residue mass by ~7%. A partially registered decrease in the residue mass during the decomposition of mixtures with DDM-DOPO can also be associated with decomposition of the additive itself in the mixture. However, the individual substance DDM-DOPO is degraded up to 90% [16]; therefore, the maximum contribution to the residue mass reduction for the ER + DDM-DOPO mixture from DDM-DOPO can be 1.3% in 3% and 2.7% in 7% for samples with 1.5% and 3% DDM-DOPO (Table 1), respectively. The addition of 1.5% graphene has practically no effect on the amount of the residue; however, 3% graphene leads to a decrease in the weight of the residue in TGA by ~7%.

**Figure 2:**
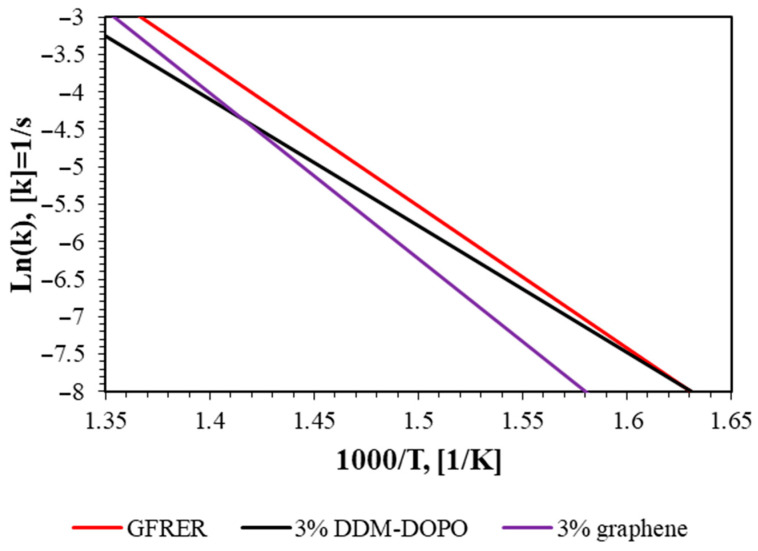
Pyrolysis rate constants of GFRER compositions in the Arrhenius form.

Assuming that the pyrolysis reaction occurs in one stage and is of the first order, from the data in Figure 1, the kinetic parameters of pyrolysis were obtained using the established method [37]. The obtained kinetic parameters for GFRER are presented in Table 2. The accuracy of determining the pyrolysis rate constant by this method is factor 5. Despite small differences in the temperature of the maximum thermal decomposition rate of the samples (Table 1), the pyrolysis kinetics barely changes with the addition of flame retardants (Figure 2) for high temperatures.

Table 1 shows UL-94 HB data with the measured average rate of spread (ROS) and the LOI test. The 50% binder GFRER failed the UL-94 test and had a burn rate of 84 mm/min (Table 1). It is to be noted that in the UL-94HB test, the speed incombustibility criterion for the HB rating for specimens up to 3 mm thick was 75 mm/min.

**Figure 3:**
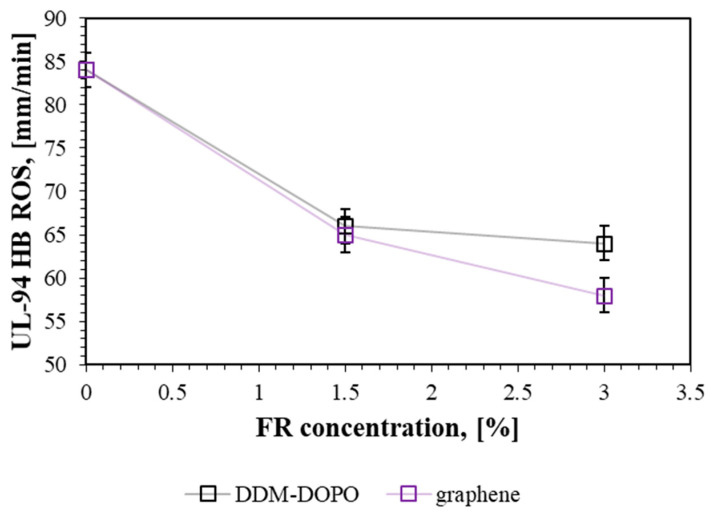
The effect of flame retardant concentration on the burning rate in the UL-94HB test.

Table 1 also shows that both flame retardants improve the fire resistance of GFRER in the UL-94 and LOI tests. At the same time, the additions of 3% DDM-DOPO and 3% graphene result in a decrease in the pyrolysis rate constant. In the UL-94 horizontal flame spread test, all flame-retardant-inhibited samples were rated as “HB” (at 1.5% and 3% additive concentrations). The addition of 1.5% DDM-DOPO to the sample reduced the flame propagation rate in the UL-94 HB test by ~27% relative to pure GFRER, while the addition of 3% DDM-DOPO reduced the rate by ~31%. Thus, a twofold increase in the additive concentration does not lead to a significant increase in the inhibition effect (the saturation effect). In the case of 1.5% graphene, the difference in the rate relative to pure GFRER was ~29%, while for 3% graphene, the difference increased to ~45%. In fact, an increase in graphene concentration leads to a noticeable increase in the inhibition effect in this test. Figure 3 shows the dependence of the UL-94HB rate of flame spread (ROS) on the concentration of flame retardant in the composition of GFRER. For graphene, a close to linear dependence of ROS on concentration is observed, and for DDM-DOPO, a decrease in efficiency occurs as the flame retardant concentration rises in the UL94 test. At the same time, at 1.5% of the additive, the effectiveness of both fire retardants is practically the same. In order to show the effectiveness of the flame retardants used, the LOI of 2 mm thick samples with a higher mass fraction of flame retardants in the composition was additionally measured. Increasing the thickness of the samples and the mass fraction of flame retardants leads to an increase in LOI, which indicates the high fire resistance of GFRER under standard atmospheric conditions. Further experiments with these samples were not carried out in this work, since it turned out to be impossible to support their combustion with an external heat flux.

### 2.2. The Flame Spread Rate over the Samples Surface

**Figure 4:**
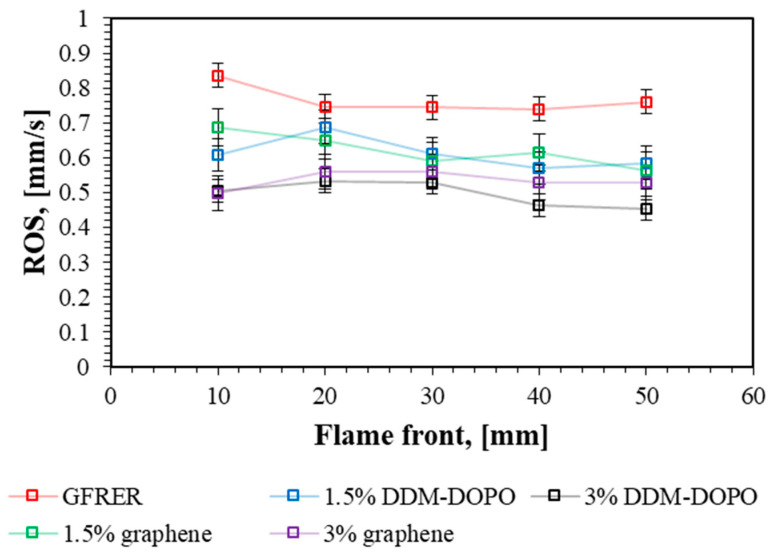
Dependence of the flame spread rate over the sample surface from the flame front position under the incident heat flux 3 kW/m^2^.

The dependence of the rate of flame spread (ROS) from a flame front position under incident heat flux (IHF) 3 kW/m^2^ is shown in Figure 4. The flame spreads over the sample surface uniformly, without acceleration. ROS fluctuations in a particular part of the sample may be connected with the inhomogeneity of the manufactured samples. The addition of the flame retardant led to a decrease in ROS over GFRER surface. It can also be noted that an increase in the flame-retardant concentration to 3% led to a greater decrease in ROS of 1.5%. The sample incorporated with 3% DDM-DOPO showed the lowest flame spread rate, although the sample incorporated with 3% graphene showed the lowest flame spread rate in the UL-94 HB test.

**Figure 5:**
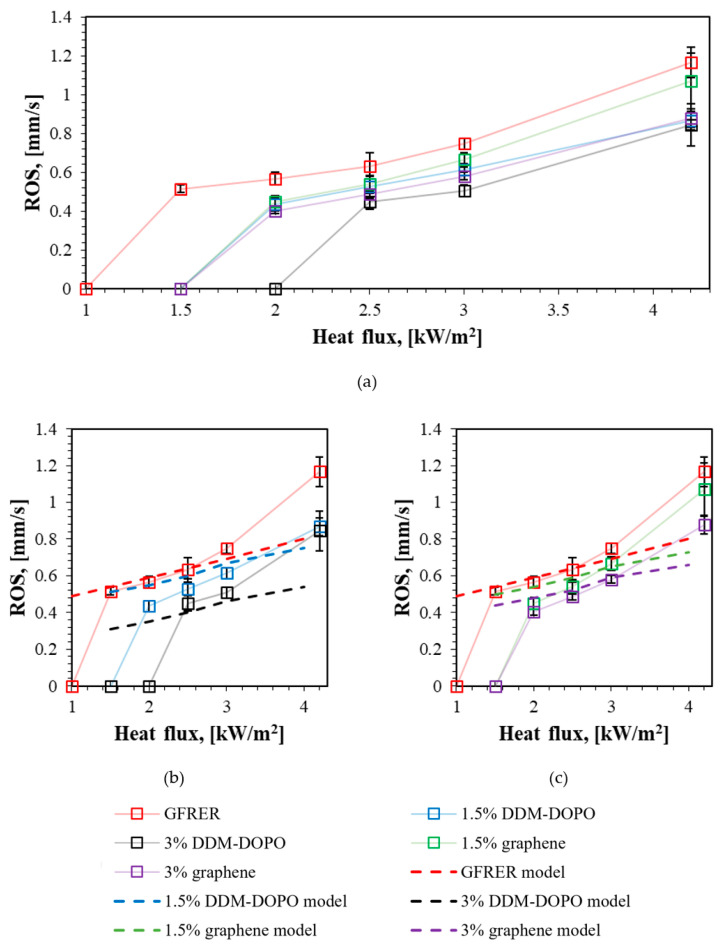
An average flame spread rate over the samples surface depending on the incident heat flux. (**a**) The experimental results. (**b**) The model and the experimental results for DDM-DOPO. (**c**) The model and the experimental results for graphene.

The dependence of the flame spread rate on different heat fluxes and flame-retardant concentrations is shown in Table 2 and Figure 5. The slope of the curves shows that the linear increase in the heat flux magnitude leads to the linear growth in ROS. The samples incorporated with 3% additive have a lower ROS compared to the samples with 1.5% additive under the heat fluxes lower than 3 kW/m^2^. The same tendency is shown in Figure 5. The sample without the additives does not demonstrate self-sustaining combustion at the external heat flux 1 kW/m^2^, the samples with 1.5% DDM-DOPO and 1.5% and 3% graphene extinguish at the external heat flux 1.5 kW/m^2^, and the sample with 3% DDM-DOPO extinguishes at the heat flux 2 kW/m^2^. When the heat flux was increased to 4.3 kW/m^2^, the ROS for GFRER sample began to deviate from the linear dependence. It can be connected with the early pyrolysis of the sample surface ahead of the flame front. Whereas the efficiency of the 1.5% graphene additive does not depend on the heat flux, the efficiency of the other additives increases when the heat flux increases. However, at the external heat flux 4.3 kW/m^2^, the efficiency of 1.5% DDM-DOPO, 3% DDM-DOPO, and 3% graphene are almost the same. In the case of DDM-DOPO, this is probably due to H and OH radicals’ equilibrium concentration in the flame. In the case of graphene, an increase in the concentration of the additive from 1.5% to 3% leads to a further decrease in ROS. The same results were obtained in UL-94 HB (Table 1), where the flame spread rate of UL-94HB for DDM-DOPO was not strongly affected by the increasing concentration, while an increase in the graphene concentration continued to reduce ROS.

The calculated ROS for GFRER and GFRER with flame retardant additives are satisfactorily predicted by the model (Figure 5b,c) described in Section 3.6. However, the flame extinguishes at a low heat flux that is not predicted by the model. Additionally, in the case of 1.5% DDM-DOPO, the model overpredicts ROS, while with 3% DDM-DOPO it slightly underpredicts ROS. This is due to the simplicity of the flame retardant action assumption in the model in the gas phase, based on the decrease in the reaction rate constant of a one-stage global reaction. The DDM-DOPO effectiveness coefficient *Ψ* in the model depends on the additive concentration, while in the experiments (Table 1, Figure 5), a decrease in the additive effectiveness with increasing concentration was observed. The results of elemental analysis obtained by scanning electron microscopy (Quantitative Energy-dispersive X-ray spectroscopy microanalysis) after the flame propagation experiments under IHF 3 kW/m^2^ for the samples incorporated with DDM-DOPO, showed that from 30 to 50% of the initial phosphorus went into the gas phase. The SEM microphotographs (Figures S1 and S2) are presented in the Supplementary Materials. All things considered, the results of elemental analysis confirm the assumption about the gas-phase mechanism inhibition of the DDM-DOPO additive. The influence of flame retardants containing phosphorus in the composition of polymeric materials was previously studied [38,39,40,41]. The main mechanism of action of these additives is believed to be participation of this species and its decomposition products in chain-termination reactions.

**Figure 6:**
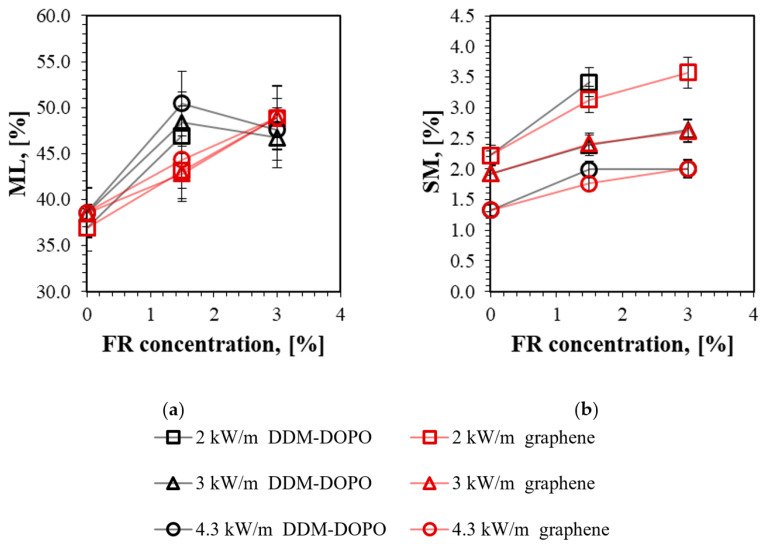
Comparison (**a**) mass loss (ML) in % of the initial sample mass depending on the additive concentration and the incident heat flux (**b**) soot mass (SM) on the sample surface in % of initial sample mass, depending on the additive concentration and the incident heat flux.

Mass loss (ML) and soot mass (SM) dependence versus flame retardant concentration and the incident heat flux obtained after the downward flame propagation experiments are shown in Figure 6. Figure 6a shows the total mass loss in % of the initial sample mass. Figure 6b shows soot mass formed during flame propagation in % of the initial sample mass.

The samples incorporated with 1.5% DDM-DOPO showed an increase in ML by ~10% compared to the sample without the additives under all the tested heat flux values. However, increasing the DDM-DOPO concentration to 3% did not affect ML within the experimental accuracy, since almost all of the binder left the sample in this case. The samples incorporated with graphene showed a linear increase in ML (5% for 1.5% graphene and 10% for 3% graphene) with increasing additive concentration for all the tested heat flux values. This is due to an increase in the gas yield for samples with additives, which was observed in the TGA (Figure 1c). It can also be seen that the heat flux magnitude has little effect on ML in the experiment.

Figure 6b shows that SM decreases for all samples with increasing heat flux. This is probably due to the more intense combustion of the sample under a higher heat flux, which leads to soot oxidation on the sample surface. An increase in the concentration of DDM-DOPO and graphene additives led to the same SM for all the cases. The addition of graphene probably leads to an increase in the proportion of non-combustible volatile pyrolysis products, which dilutes combustible gases and extinguishes the flame. DDM-DOPO decomposes under the action of temperature and its phosphorus-containing decomposition products act in the gas phase. In this case, the effect of reducing the efficiency of DDM-DOPO with an increase in its initial concentration in the polymer was observed. An increase in the soot yield for the samples incorporated with graphene compared to the samples without the additives and samples incorporated with DDM-DOPO under elevated oxygen concentration conditions was observed in work [22].

### 2.3. Thermal Flame Structure

**Figure 7:**
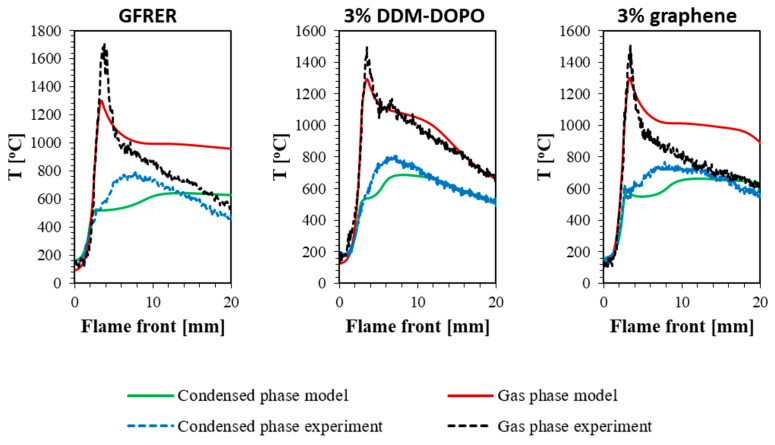
Comparison of the temperature profiles in the condensed phase and in the gas phase at the 1 mm distance from the sample surface under the incident heat flux 3 kW/m^2^, obtained from the experiment and from the model.

**Figure 8:**
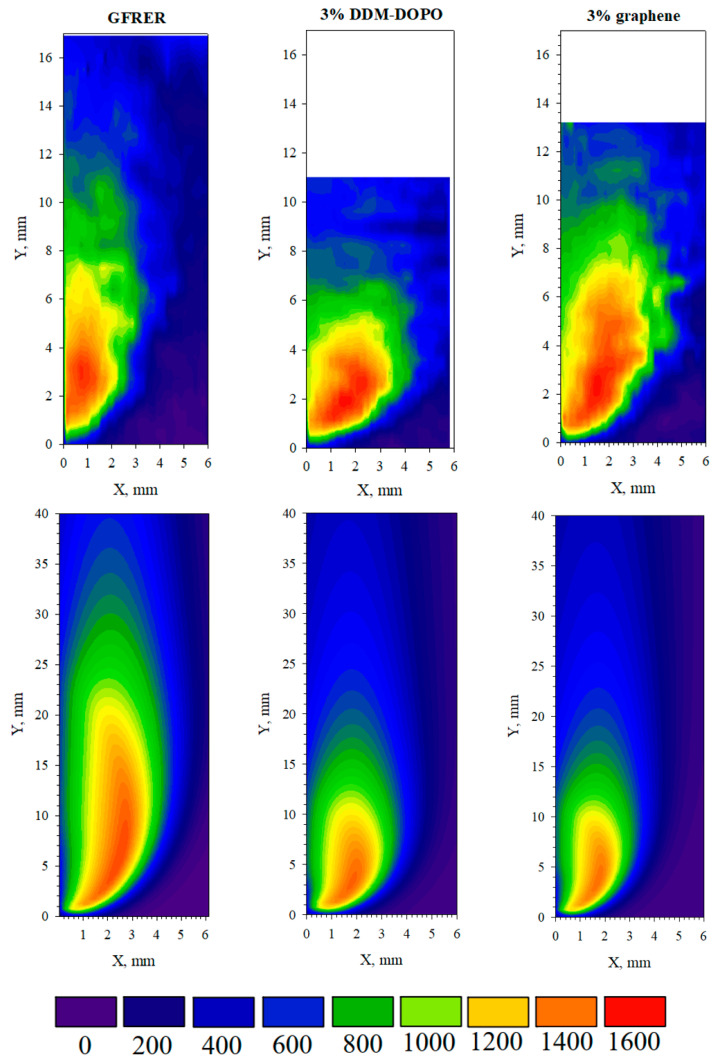
Comparison of the thermal flame structure in the experiment and in the model under the incident heat flux 3 kW/m^2^.

The temperature profiles in the gas and condensed phases obtained in the experiment and the model are compared in Figure 7. The flame structures obtained by scanning with a thermocouple and the flame structure obtained from the model are shown in Figure 8. The temperature profile gradients for the condensed phase in the preheating zone show good agreement between the experiment and the model. Yet, the major contribution to the rate of flame front shifting (ROS) is made by the temperature gradient from the flame to the sample surface near the flame front, where, as the observations have shown, there is no soot accumulation; therefore, the ROS is well predicted by the model. The maximum surface temperature in the model has a lower value regarding the experiment due to the underestimation of soot formation on the surface. In the region after the maximum, the model better predicts the temperature profile for samples with 3% DDM-DOPO and 3% graphene additives, while for the GFRER sample, the temperature in the model is overestimated. Thus, the model satisfactorily predicts the width of the combustion zone for samples with additives.

Figure 7 shows the comparison of the temperature profiles in the gas phase at a distance of 1 mm from the sample surface obtained in the model and in the experiment. In the gas phase, the model also predicts the temperature profile gradient well, but underestimates the maximum temperature. The maximum temperature in the gas phase for all the samples was approximately 1600–1650 °C (Figure 8) within the thermocouple measurement error (±50 °C). However, for GFRER without the additives, the flame was pressed against the sample surface strongly; therefore, the maximum temperature recorded for GFRER at 1 mm distance from the surface (Figure 7) was higher than for the samples with 3% DDM-DOPO and 3% graphene. In the model, the maximum temperature in the gas phase at a distance of 1 mm from the surface was underestimated. Figure 8 shows that this is due to the fact that in the model the flame is not so strongly pressed against the surface and the zone of the maximum temperature is located slightly higher. This deviation is connected with a single-stage global macro reaction of combustion, because in the experiment near the combustion surface, many thermal decomposition reactions and the combustion of the volatile pyrolysis products of epoxy resin take place [42], which are not considered in the model. At the same time, volatile combustible products only appear on the sample surface in the model.

The flame height was determined as its projection onto the sample plane from the beginning of the flame front to the region of 800 °C. The flame height for GFRER without the additives was 12 mm. The addition of the flame retardants reduced the length of the luminous flame zone by 4 mm in the case of DDM-DOPO and had almost no effect in the case of graphene. In the case of 3% graphene, the model reduced the length of the luminous flame zone and made it comparable in length with the experiment; however, the model incorrectly reproduced the trend that was observed in the experiment, where the addition of 3% graphene had almost no effect on the length of the luminous flame zone. It is to be emphasized that in the DDM-DOPO case, the model also reduced the length of the luminous flame zone and made it closer to the experiment, and, more importantly, the model decreased the luminous flame zone compared to pure GFRER observed in the experiment. This can explain the underprediction of the temperature values by the model in the gaseous and condensed phases (Figure 7) after passing the maximum on the temperature profiles GFRER without the additives and GFRER incorporated with 3% graphene and, consequently, it can explain the good temperature value agreement for 3% DDM-DOPO. Finally, it can be concluded that the use of a one-step inhibition mechanism for DDM-DOPO in the gas phase, connected with a decrease in the pre-exponential coefficient for the burning rate in the gas phase k_g_, allows one to predict the DDM-DOPO fire-retardant effect on the flame behavior for GFRER with satisfactory accuracy. On the other hand, the mechanism of the graphene action, which is considered to force some of the volatile pyrolysis products to become non-combustible and not to participate in the combustion, requires more detailed study and the refinement of the model.

## 3. Materials and Methods

### 3.1. Sample Preparation

#### 3.1.1. Epoxy Matrix

For glass fiber-reinforced epoxy-resin preparation, a polymer matrix was prepared with the composition presented in Table 3. ED-22 is a commercially available epoxy resin. Graphene and DDM-DOPO were used as flame retardants (9,10-dihydro-9-hydroxy-10-phosphaphenantrene-10-oxide-diaminodiphenylmethane).

Before being introduced, graphene was dispersed in an ultrasonic bath in acetone in the ratio of 1 g graphene to 100 mL acetone for 1 h at room temperature. After that, epoxy resin was added to the graphene dispersion in acetone and was stirred for 2 h. Then, the mixture was heated in an oil bath at the temperature of 130 °C during 0.5 h to remove acetone [43]. After that, the mixture was cooled to 50 °C, finely ground DDM powder was added to it, and the mixture was stirred for 2 h. Before DDM-DOPO and the curing agent (DDM) were introduced into epoxy resin, they were ground to fine powder and then mixed with the resin during 2 h at 50 °C. The obtained mixtures of resin, graphene and DDM, as well as of resin, DDM-DOPO and DDM, were used for making prepregs. The glass-fiber matrix was oriented in one direction for all the layers of the prepreg based on T-15(P)-76(92) glass fabric, indicating the one-directional structure of the fiber reinforcement. The curing mode was as follows: 100 °C for 2 h, 150 °C for 2 h. There were 2 layers in the glass fiber fabric. The binder content in the prepreg was 50%mass. Densities of the samples with compositions 1–5 were 1.44–1.55 g/cm^3^ and with compositions 6–7 were ~1.1 g/cm^3^.

For samples 6 and 7 (thickness ~2 mm), flame-propagation experiments were not carried out due to the low combustibility of these materials (they do not support flame spread under the action of a heat flux created by the radiation panels used in the work).

#### 3.1.2. Additives

DDM-DOPO was provided for the tests by Prof. Yuan Hu from the USTC China. The method of synthesizing DDM-DOPO was previously described in [16].

Graphene was produced by the RUSGRAPHENE company (https://rusgraphene.com/, accessed on 1 June 2023).

### 3.2. Thermal Degradation Analysis

The thermal decomposition of the samples was studied using TGA. Pieces of GFRER slabs weighing 3–4 mg were placed into an aluminum or platinum crucible using a synchronous TG/DSC analyzer STA 409 PC (Netzsch, Selb, Germany) in a 100 v% argon and 79 v% He + 21 v% O_2_ flow with a volumetric velocity of 27 cm^3^/min (NTP). The samples were heated from 30 to 580 °C at the heating rate of 30 K/min. All the experiments were repeated at least 3 times. The accuracy of determining the decomposition residue was ~3%.

### 3.3. Elemental Analysis

Elemental analysis of the samples with the addition of DDM-DOPO to test the presence of phosphorus on the surface before and after combustion in the experiment, under the action of external radiation, was carried out using a JSM-6460LV SEM (JEOL, Tokyo, Japan) microscope (using Quantitative Energy-dispersive X-ray spectroscopy microanalysis).

### 3.4. Flammability Tests

The accuracy of determining the burning velocity in UL-94HB test was ±3–5%. The limiting oxygen index test was carried out in accordance with ISO 4589-2. The accuracy of the LOI was ±0.1%.

### 3.5. Description of the Experimental Setup for Downward Flame Propagation under the Action of Bilateral External Heating

**Figure 9:**
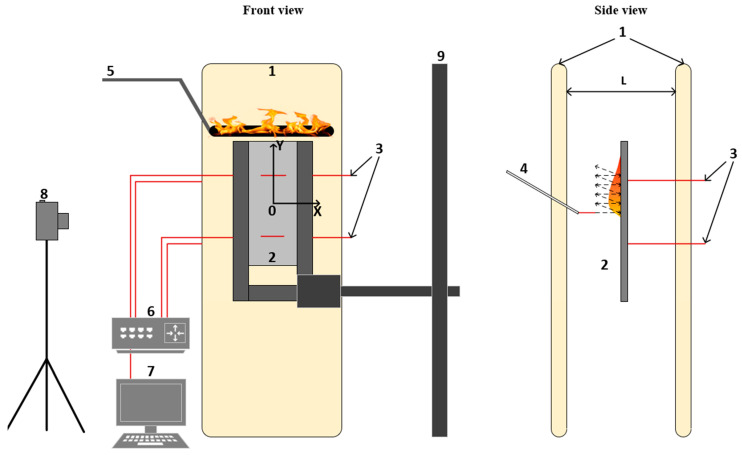
The configuration of the experimental setup for downward flame propagation under the action of a heater. 1—heaters, 2—sample in an aluminum frame, 3—thermocouples on the sample surface and in the gas phase, 4—scanning thermocouple, 5—ignition torch, 6—ADC, 7—PC, 8—camera, 9—sample holder.

The experiment on downward flame propagation under the action of two heaters was carried out on the setup shown in Figure 9.

The sample dimensions were 75 × 25 × 0.4 mm^3^. The sample was installed in an aluminum frame 1 mm thick to prevent the flame spread along the side surfaces and to limit the width of the combustion zone to 20 mm. Using a tripod, the sample was fixed between two heaters (Almac IK5 Infrared Electric Heater, 500 Watt) powered by 220 V AC. The value of the incident heat flux (IHF) to the sample surface, depending on the distance from the heater from 25 to 100 mm, varied from 1 to 3 kW/m^2^. The heat flux of 4.2 kW/m^2^ was obtained by increasing the power supply voltage to 260 V. The sample was placed between two heaters at an equal distance from its surface. Calibration of the heating panels was previously carried out using the heat flux sensor described in [44]. The heat flux setting accuracy was ±0.3 kW/m^2^. After installing the sample, the heating panels were switched on and heated for 15 min until a constant heat flux was established. To form a uniform flame front, the sample was ignited using a torch soaked in alcohol, which covered the entire length of the upper face of the sample. The sample was marked by horizontal lines (every 10 mm). The rate of flame spread over the surface of the sample was determined by the position of the luminous flame front, determined by video recording with a FujiFilm x-A20 (Fujifilm Holdings Corporation, Tokyo, Japan) camcorder (the shooting frequency was 30 frames per second). The temperature on the sample surface and in the gas phase was measured with a Pt-Pt + 10% Rh thermocouple (S-type thermocouple, wire diameter was 50 µm thick) [24]. The radiation correction was taken into account using the formula [45]. To determine the thermal flame structure, a Pt-Pt + 10% Rh thermocouple was used, coated with a layer of SiO_2_ to prevent catalytic reactions on the surface of the thermocouple. The thermocouple was mounted on a biaxial positioning system with two stepper motors. In the initial position, the thermocouple junction was placed at the center of the sample with respect to the X axis at a distance of 6 mm from the sample plane. The thermocouple moved to the surface of the sample to the surface with a horizontal speed of 1 mm/s and a vertical speed equal to the speed of the flame. Flame scanning was carried out with a step of 1 mm along the Y axis. All the thermocouples were connected to a multi-channel 14 bit analog to digital converter (ADC) E14-140M, which was connected to a PC. To determine the weight loss of the samples, as well as the amount of soot on the surface of the burnt sample, additional experiments were carried out without connecting thermocouples. After the burning of the sample, soot was collected from its surface, and its weight was measured. The complete yield of the combustion products into the gas phase, including gaseous volatile products and soot deposits, was determined as the difference in mass between the original sample and the burnt sample, which was normalized to the mass of the original sample.

### 3.6. Numerical Approach

The following mathematical model was proposed to predict a behavior of flame spread over polymers combined with flame retardants. The mathematical model takes into account coupled heat and mass transfer between gas phase flame and solid fuel, multicomponent reacting gas flow, gas phase combustion, heat transfer and pyrolysis in a solid material [22,24,36]:

(1) $$\frac{\partial \rho }{\partial t}+\frac{\partial \rho u_{j}}{\partial x_{j}}=0,$$

(2) $$\rho \frac{\partial u_{i}}{\partial t}+\rho u_{j}\frac{\partial u_{i}}{\partial x_{j}}=-\frac{\partial p}{\partial x_{i}}+\frac{\partial }{\partial x_{j}}\mu \frac{\partial u_{i}}{\partial x_{j}}+\left(\rho_{a}-\rho \right)g_{i},$$

(3) $$\rho C\frac{\partial T}{\partial t}+\rho u_{j}C\frac{\partial T}{\partial x_{j}}=\frac{\partial }{\partial x_{j}}\lambda \frac{\partial T}{\partial x_{j}}+\rho WQ-\frac{\partial q_{j}^{r}}{\partial x_{j}},$$

(4) $$\rho \frac{\partial Y_{k}}{\partial t}+\rho u_{j}\frac{\partial Y_{k}}{\partial x_{j}}=\frac{\partial }{\partial x_{j}}\rho D\frac{\partial Y_{k}}{\partial x_{j}}+\nu_{k}\rho W,$$

(5) ρ=p/RT.

Here $x_{i}=\left\{x,y\right\}$, $u_{i}=\left\{u,v\right\}$, k={F,O,P}, $\nu_{k}=\left\{-1,-\nu_{O},1+\nu_{O}\right\}$, $g_{i}=\left\{g,0\right\}$.

A single-step mechanism is employed here for a gas phase combustion reaction as follows

(6) $$F+\nu_{O}O+I\to \left(1+\nu_{O}\right)P+I,$$

in which reaction rate is expressed in an Arrhenius form as

(7) $$W=kY_{F}Y_{O}\exp\left(-E/R_{0}T\right).$$

The model of a solid material is extended to resolve the thermal degradation of the composite material. Such a composite consists of a combustible part (binder) reinforced with a noncombustible (e.g., glass fibers). The model was formulated to take into account these two components. Also, the model takes into account the anisotropy of thermal conductivity of a solid material. The energy conservation equation of solid material was expressed as:

(8) $$\rho_{s}C_{s}\frac{\partial T_{s}}{\partial t}=\frac{\partial }{\partial x_{j}}\lambda_{s}^{j}\frac{\partial T_{s}}{\partial x_{j}}+\eta_{b}^{0}\rho_{b}Q_{b}W_{b},$$

The reaction rate of the pyrolysis reaction is given by

(9) $$W_{b}={\left(1-\alpha \right)}^{n}k_{b}\exp\left(-E_{b}/R_{0}T_{s}\right),$$

where the conversion degree varies from 0 to 1 and is defined as

(10) $$\frac{d\alpha }{dt}=W_{b}.$$

The density of the solid material was defined taking into account binder burnout as follows:

(11) $$\rho_{s}=\eta_{b}^{0}\left(1-\alpha \right)\rho_{b}+\left(1-\eta_{b}^{0}\right)\rho_{f},$$

The local mass burning rate (combustible volatiles gasification rate) at a burning surface was defined as

(12) $${\dot{m}}_{b}=\eta_{b}^{0}\rho_{b}\int_{-L_{s}}^{0}W_{b}dy.$$

According to the previous studies [22,36], a DOPO-based flame retardant was considered to have an effect in the gas phase. Thus, the pre-exponential factor of the gas phase combustion reaction is reduced in the following way:

(13) $$k_{g,DOPO}=\left(1-\psi_{DOPO}Y_{DOPO}\right)k_{g},$$

where $Y_{DOPO}$ is the initial mass fraction of DDM-DOPO in the solid material, and $\psi_{DOPO}$ is the DOPO inhibition effect coefficient.

A graphene-based flame retardant was proposed [22] to have an effect by a reduction in the amount of gaseous pyrolysates available for combustion in the following way. Only the $\psi_{gr}Y_{gr}{\dot{m}}_{b}$ part of the local mass burning rate given by Equation (12) was set to go to the gaseous fuel (*F*), while $\left(1-\psi_{gr}Y_{gr}\right){\dot{m}}_{b}$ part supplied a non-combustible gas, which was set to be the products (*P*). Here, $\psi_{gr}$ represents the inhibition effect coefficient of graphene, and $Y_{gr}$ is the graphene mass fraction.

**Figure 10:**
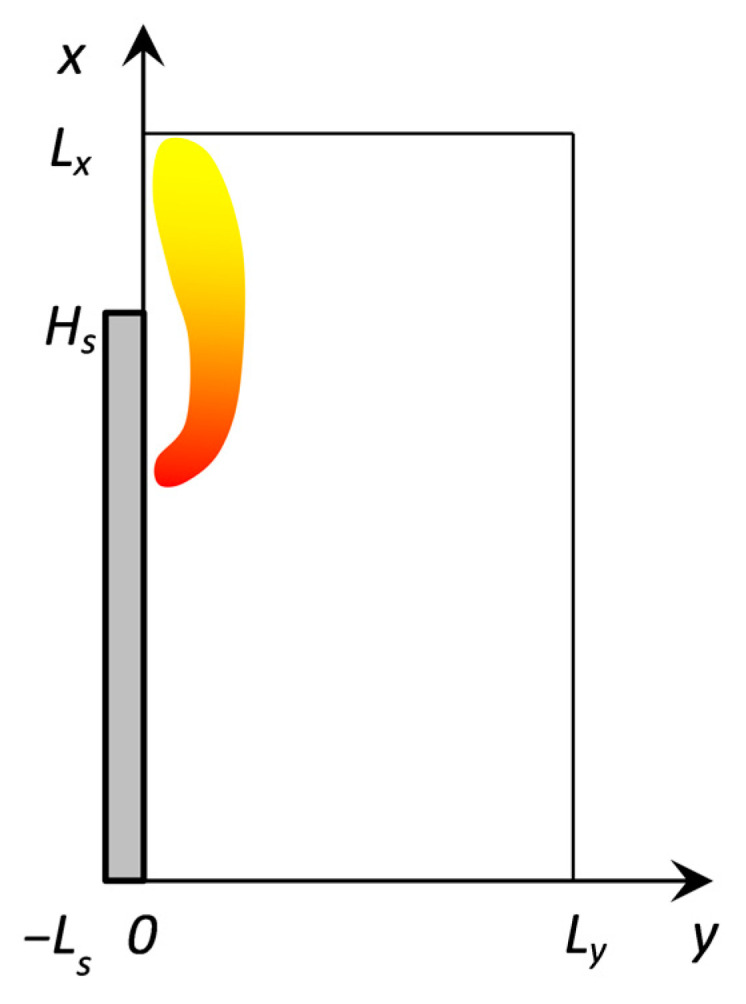
Arrangement of the computation domain.

The boundary conditions were set according to the computational domain scheme (Figure 10) as follows:

x=0:$T=T_{a}$, $Y_{O}=Y_{O,a}$, $Y_{F}=0$, $Y_{P}=0$, ∂u/∂x=0, v=0;$x=L_{x}$:∂ϕ/∂x=0, $\phi =\left\{u,v,T,Y_{k}\right\}$, k={F,O,P};$y=L_{y}$:$T=T_{a}$, $Y_{O}=Y_{O,a}$, $Y_{F}=0$, $Y_{P}=0$, u=0, ∂v/∂y=0;y=0, $H_{s}<x<L_{x}$:v=0, ∂ϕ/∂y=0, $\phi =\left\{u,T,Y_{k}\right\}$, k={F,O,P};$y=-L_{s}$, $0<x<H_{s}$ x=0, $-L_{s}<y<0$: $x=H_{s}$, $-L_{s}<y<0$$\partial T_{s}/\partial n=0$;y=0, $0<x<H_{s}$:u=0, $\rho v=\rho_{s}u_{s}$, $T_{s}=T$, $-\lambda_{s}\frac{\partial T_{s}}{\partial y}+{\left(\rho uCT\right)}_{s}=-\lambda \frac{\partial T}{\partial y}+\rho vCT+q_{w}^{r}+q_{ext}$, $-\rho D\frac{\partial Y_{F}}{\partial y}+\rho vY_{F}=\rho_{s}u_{s}$, $-\rho D\frac{\partial Y_{k}}{\partial y}+\rho vY_{k}=0$, k={O,P};

Table 4 presents the inhibition effect values in the gas phase used in the model and the kinetic parameters of sample pyrolysis determined from TGA data. In the model for samples with flame retardants, the pyrolysis kinetics determined for the maximum concentration of the additive (3%) was used, since the effect of the additives on the thermal decomposition rate constant was relatively small.

## 4. Conclusions

An experimental and numerical study of the DDM-DOPO-and-graphene flame retardant’s impact on the flammability of the epoxy resin-based composites was carried out using both traditional flammability tests (UL-94, LOI), thermogravimetry and a downward flame propagation study under the action of bilateral external heating. For the 2 mm thick samples incorporated with 9.8% DDM-DOPO, the LOI was 26.4%; therefore, this composition can be applied in practice. For the first time, using thin thermocouples, the thermal structure of the downward spreading flame of fiberglass-reinforced (~50 wt.%) epoxy resin thin samples (~0.4 mm) with and without the flame retardants DDM-DOPO and graphene (1.5 and 3 wt.%) were measured in a quiescent air atmosphere under the action of an additional heat flux (up to 4.2 kW/m^2^). For the first time, the measured thermal flame structure was compared with the numerical simulation results of the flame propagation over the GFRER surface. The flame retardants were shown to be effective (a decrease in the flame spread rate) in improving the fire resistance of the epoxy resin composites. The flame spread over the surface of the sample almost uniformly, without acceleration. The flame spread rate had an almost linear dependence on the heat flux, which was well predicted by the model (up to 3 kW/m^2^). As the external heat flux increased, an increase in the flame spread rate was observed, which was associated with the approach of the sample surface temperature to the epoxy resin pyrolysis temperature. Under the heat flux 4.2 kW/m^2^, an increase in the additive concentration from 1.5% to 3% for DDM-DOPO had almost no effect on the flame spread rate. A similar result was observed in UL-94 HB, where the flame propagation rate did not change with increasing DDM-DOPO concentration. Also, an increase in the DDM-DOPO concentration had almost no effect on the mass consumption during downward flame propagation.

The numerical model of the downward flame propagation under the action of bilateral external heating over the fiberglass-reinforced epoxy resins with and without the flame retardants DDM-DOPO and graphene was developed. The numerical model well predicted the temperature gradient in the condensed and gaseous phases, whereby the model well predicted the flame spread rate over the samples surface. It should be emphasized, the effectiveness of DDM-DOPO in the experiment decreases with increasing flame-retardant concentration, while in the model, the effectiveness of DDM-DOPO was linear. This is due to the linear dependence of the burning rate in the gas phase from the flame-retardant concentration in the model. The comparison of the flame structure from the model and from the experiment showed that the model for DDM-DOPO predicted the trend towards a decrease in the length of the luminous flame zone well. In the case of graphene, the model incorrectly predicted the trend, reducing the length of the luminous zone; however, it correctly described the decrease in the flame spread rate and the surface temperature. In the model, the DDM-DOPO inhibition mechanism was limited by a one-step reaction, and graphene worked only at the solid material boundary, reducing the proportion of combustible pyrolysis products. Such simplicity of the mechanism inevitably entails limitations and certain disagreements with the experiment, and, of course, using a more detailed inhibition mechanism for DDM-DOPO or graphene will result in a better agreement with the experiment. The obtained data may be used for designing effective reinforced non-combustible composites applied in the aircraft industry and for determining a detailed mechanism of the effect of flame-retardant additives.

## Figures and Tables

**Table 1:** UL-94, LOI, mass loss (ML) and soot mass (SM) in downward flame-spread experiment, max T and residue mass in TGA.

Sample	UL-94 HB, with ROS mm/min	LOI, % Thin Samples	ML in % of the Initial Mass	SM in % of the Initial Mass	T_max_ DTG in Inert, °C	Residue Mass in TGA, %
GFRER	84 ± 2 ^1^	21.0	18	12	442 ± 2	59.1 ± 1.5
1.5% DDM-DOPO	66 ± 2 ^2^	21.9	14	17	438 ± 2	56.5 ± 1.5
3% DDM-DOPO	64 ± 2 ^2^	21.9	13	20	444 ± 2	55.0 ± 1.5
1.5% graphene	65 ± 2 ^2^	21.7	13	18	442 ± 2	59.1 ± 1.5
3% graphene	58 ± 2 ^2^	21.6	11	23	448 ± 2	53.7 ± 0.5
6.5% DDM-DOPO	23 ± 1 ^2^	26.1	-	-	-	-
9.8% DDM-DOPO	26 ± 1 ^2^	26.4	-	-	-	-

^1^ no rating; ^2^ HB rating.

**Table 2:** The measured flame spread rate depending on the magnitude of the heat flux and the concentration of flame retardants.

Heat Flux, kW/m^2^	Samples				
	GFRER	1.5% DDM-DOPO	3% DDM-DOPO	1.5% Graphene	3% Graphene
4.2	1.17 ± 0.08	0.87 ± 0.05	0.85 ± 0.1	1.07 ± 0.14	0.88 ± 0.05
3	0.75 ± 0.03	0.61 ± 0.03	0.51 ± 0.03	0.67 ± 0.04	0.58 ± 0.02
2.5	0.63 ± 0.07	0.53 ± 0.05	0.45 ± 0.04	0.54 ± 0.04	0.49 ± 0.02
2	0.57 ± 0.03	0.43 ± 0.03	Self-extinguished	0.45 ± 0.03	0.40 ± 0.02
1.5	0.52 ± 0.02	Self-extinguished	Self-extinguished	Self-extinguished	Self-extinguished

**Table 3:** Epoxy matrix composition.

Component	Composition, Mass Fraction						
	1	2	3	4	5	6	7
Epoxy resin ED-22	100	100	100	100	100	100	100
Curing agent #9 [24]	5	5	5	5	5	13	13
DDM-DOPO	-	1.5	3	-	-	6.5	9.8
Graphene	-	-	-	1.5	3	-	-

**Table 4:** Inhibitor efficiency parameters in the gas phase (determined in the model) and kinetic parameters of the pyrolysis rate constant in a one-stage approximation (determined experimentally).

	*Ψ*	*k_b_*, 1/s	*E_b_*, kJ/mole
GFRER	0	10^9.93^	157.3
GFRER + DDM-DOPO	29	10^8.49^	140.4
GFRER + graphene	2/3	10^11.68^	183.5

## Data Availability

Not applicable.

## Supplementary Materials

The following supporting information can be downloaded at: https://www.mdpi.com/article/10.3390/molecules28135162/s1, Figure S1: SEM micrographs and X-ray energy dispersive spectra for glass fiber reinforced epoxy resin samples with 1.5% DOPO-DDM additives, Figure S2: SEM micrographs and X-ray energy dispersive spectra for glass fiber reinforced epoxy resin samples with 3% DOPO-DDM additives.
