# Supplementary material for: The Effect of Flame-Retardant Additives DDM-DOPO and Graphene on Flame Propagation over Glass-Fiber-Reinforced Epoxy Resin under the Influence of External Thermal Radiation
Source: Molecules. 2023 Jul 1;28(13):5162. doi: 10.3390/molecules28135162 (PMC10343289; doi:10.3390/molecules28135162)
Supplement: Supplementary file 1 [file molecules-28-05162-s001.zip › molecules-2444811-supplementary.pdf]

The effect of flame retardant additives DDM-DOPO and graphene on flame propagation over glass fiber-reinforced epoxy resin under the influence of external thermal radiation

O.P Korobeinichev[1,*], E.A. Sosnin[1,2], A.A. Shaklein[3], A.I. Karpov[3], S.A. Trubachev[1], A.G. Shmakov[1], A.A. Paletsky[1], A.R. Sagitov[1,2], I.V. Kulikov[1]

[1] Voevodsky Institute of Chemical Kinetics and Combustion SB RAS, 630090 Novosibirsk, Russia
[2] Department of Physics, Novosibirsk State University, 630090 Novosibirsk, Russia
[3] Udmurt Federal Research Center, 426067 Izhevsk, Russia
*Corresponding author: okorobeinichev@gmail.com

Supplementary material

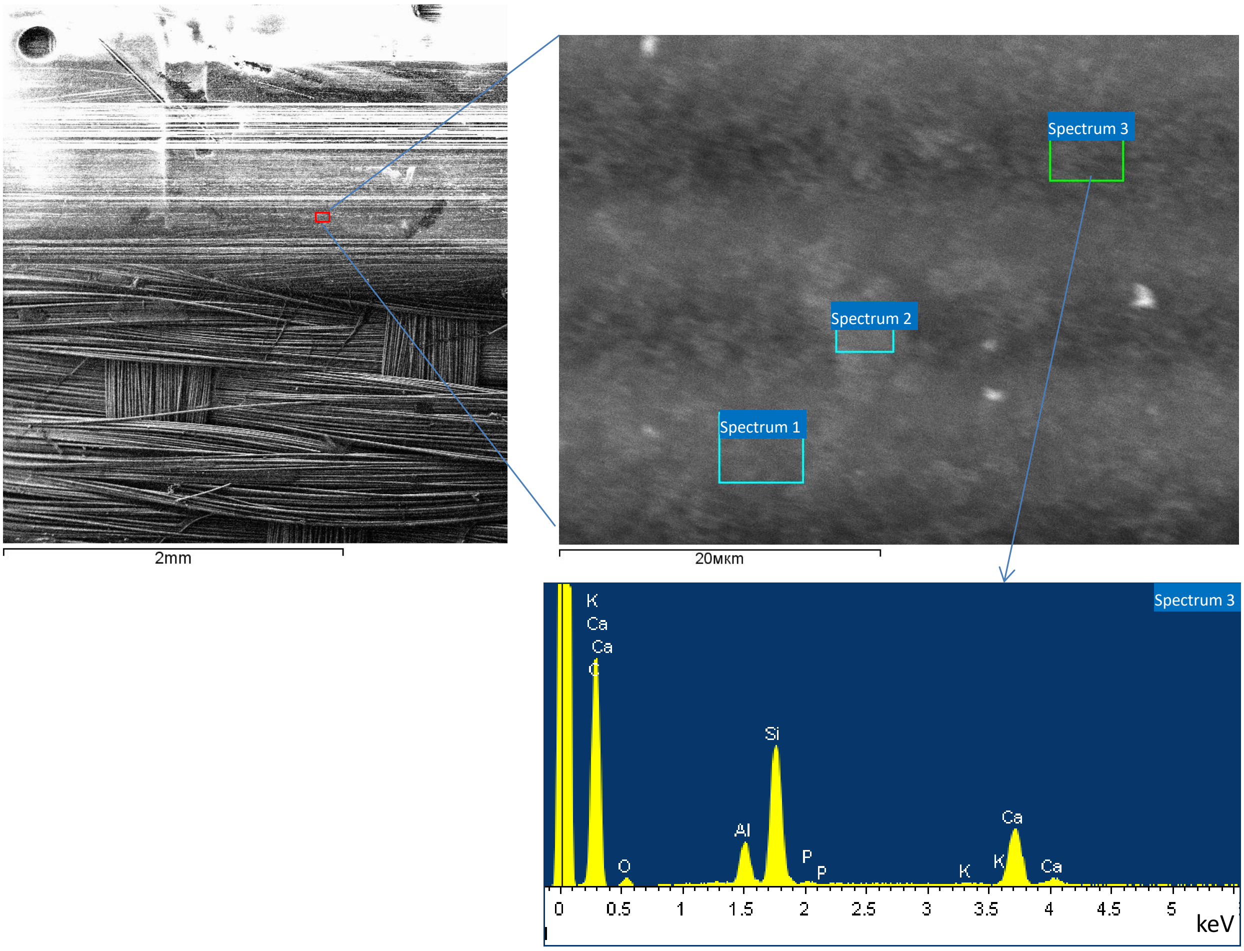

Figure S1. SEM micrographs and X-ray energy dispersive spectra for glass fiber reinforced epoxy resin samples with 1.5% DOPO-DDM additives.

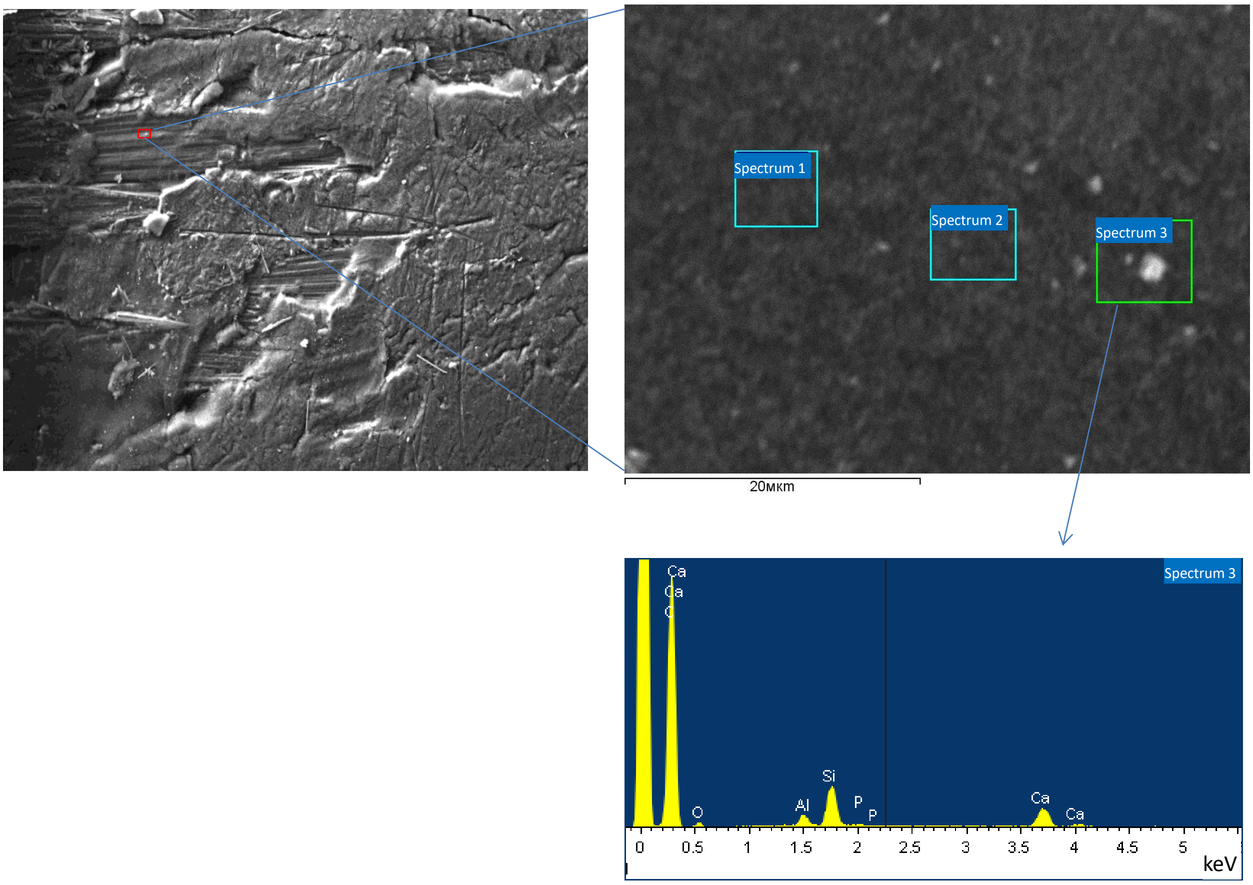

Figure S2. SEM micrographs and X-ray energy dispersive spectra for glass fiber reinforced epoxy resin samples with 3% DOPO-DDM additives.
